# Clinical and Microbiological Effects of Photodynamic Therapy Associated with Non-surgical Treatment in Aggressive Periodontitis

**DOI:** 10.5681/joddd.2014.028

**Published:** 2014-09-17

**Authors:** Mohammad Taghi Chitsazi, Adileh Shirmohammadi, Reza Pourabbas, Nader Abolfazli, Ilnaz Farhoudi, Behrouz Daghigh Azar, Farrokh Farhadi

**Affiliations:** ^1^Associate Professor, Department of Periodontics, Dental and Periodontal Research Center, Tabriz University of Medical Sciences, Tabriz, Iran; ^2^Professor, Department of Periodontics, Dental and Periodontal Research Center, Tabriz University of Medical Sciences, Tabriz, Iran; ^3^Postgraduate Student, Department of Periodontics, Tabriz University of Medical Sciences, Tabriz, Iran; ^4^Master of Science, Head of DANESH Molecular Pathology Laboratory, Tarbiz, Iran; ^5^Assistant Professor, Department of Oral and Maxillofacial Surgery, Faculty of Dentistry, Tabriz University of Medical Sciences, Tabriz, Iran

**Keywords:** Aggressive periodontitis, photochemotherapy, therapy

## Abstract

***Background and aims.*** The aim of this study was to compare the effectiveness of adjunctive photodynamic therapy (PDT) in the treatment of aggressive periodontitis.

***Materials and methods.*** A total of 24 patients with clinical diagnosis of aggressive periodontitis received scaling and root planing (SRP) for periodontal treatment. In a split-mouth design study, the teeth of one quadrant of each arch with ≥4 mm of probing depth were selected randomly for additional treatment with PDT (test group). PDT was performed with a diode laser beam with a wavelength of 670-690 nm and a power of 75 Mw. The control group consisted of selected teeth of the contralateral quadrant (SRP only). Before any treatment, subgingival plaque samples were collected by an endodontic paper cone for microbiological analysis by real-time polymerase chain reaction (PCR) for detection of *Aggregatibacter actinomycetecommitans.* Clinical parameters including clinical attachment loss (CAL) as primary outcome, plaque index (PI), bleeding on probing (BOP), probing depth (PD) and gingival recession (REC) were measured at baseline and after 90 days. Inter-group and intra-group statistical analyses were performed.

***Results.*** Treatment groups showed an improvement in all the clinical parameters and a significant reduction in the counts of *A. actinomycetecommitans* at 90 days compared to baseline (P < 0.05). None of the periodontal parameters exhibited significant differences between the two groups (P > 0.05).

***Conclusion.*** Within the limitations of this study, the results did not show additional benefits from PDT as an adjunctive treatment for patients with aggressive periodontitis.

## Introduction

Periodontitis is one of the major causes of tooth loss and is principally caused by bacterial infection.^1,2^ Scaling and root planing (SRP) is the most effective path to eliminate the cause of this disease. The treatment of aggressive periodontitis has always been a challenge for clinicians and there are no established protocols or guidelines for the effective treatment of this disease.^3^ It is clearly established that inhibition of progression of this disease is based on reduction or elimination of periodontopathogenic bacteria. Except for common SRP and surgical treatment methods, various adjunctive treatments have been introduced in order to sufficiently eliminate microorganisms from the periodontal pocket.^4,5^ Photodynamic therapy might become a method of adjunctive treatment for aggressive periodontitis.

Photodynamic therapy (PDT) was introduced in medical therapy for inactivation of microorganisms on the basis of photosensitizer attachment to target cells. PDT can be activated by a suitable wavelength of light,^6^ but even broad-spectrum light can activate photosensitizers such as toluidine blue.^7,8^ Established photosensitizers such as toluidine blue have been reported to be antibacterial, antiviral and antiprotozoal since World War II.^9^ Toluidine blue has been shown to be highly effective when used with a soft laser irradiation.^10-12^

Many studies have shown that a highly virulence factor is associated with aggressive periodontitis.^13,14^ *Aggregatibacter actinomycetemcomitans* is frequently detected in patients with localized aggressive periodontitis, whereas in generalized forms of aggressive periodontitis, which affects most of the dentition, a different microbiota, including *Porphyromonas gingivalis* and *Tannerella forsythensis* (formerly *Bacteroides forsythus*) has been suggested.^13,15-18^

One of the most obvious associations between a suspected pathogen and periodontitis is seen with *A. actinomycetecommitans,* which is a small, non-motile, gram-negative, capnophilic rod. This species was first recognized as a periodontal pathogen by its increased frequency of detection and higher counts in lesions of localized aggressive periodontitis,^19^ when compared to counts in plaque samples of infected teeth. In the clinical conditions when subjects with this form of disease were treated successfully, the species was eliminated or lowered in counts; treatment failures were associated with failure to decrease the number of the species in treated sites.^20,21^

An ideal method of counting should be able to quantify multiple species and be sensitive and specific. Quantity is essential because the difference in the microbial species between periodontal health and disease and between pre- and post-periodontal treatment is quantitative rather than presence or absence of one or more species of a pathogen. Many studies have revealed a qualitative relationship between the presence of periodontopathogenic bacteria and pocket depth.^1,22,23^ PCR is a common method capable of detecting low number of cells but it is not able to provide quantitative data. Real-time PCR overcomes this limitation.^24^ Real-time PCR assay for periodontal bacteria can be used to determine bacterial counts for a wide range of purposes in the study of periodontal diseases.^25^

Data from in vitro studies have shown that several periodontal pathogens, such as *Porphyromonas gingivalis* and *A. actinomycetecommitans* are efficiently eliminated by PDT, either in the aqueous suspension or as biofilm. Moreover, in vitro studies may not reflect the situation of the oral environment that could potentially interfere with PDT effectiveness, such as serum or blood, both of which would be found in gingival sulcus or periodontal pockets.^8^

However, to the best of our knowledge, data from clinical trials evaluating the effect of PDT are, currently, still limited. Moreover, there is little data available on the clinical and microbiological effect of PDT in the treatment of aggressive periodontitis.

Therefore, the aim of this study was to evaluate clinical and microbiological effectiveness of adjunctive PDT in non-surgical periodontal treatment of patients with aggressive periodontitis.

## Materials and Methods

For calculation of sample size the study of Panos^5^ was used. It included an a error of 5%, 80% power; an SD of 0.07 mm of clinical attachment level (primary outcome) and a difference of 0.2 mm between the groups were considered clinically significant. It was indicated that a sample of 12 patients per group would be needed.

Therefore, 24 patients (15 females, 9 males, with a mean age of 29) with clinically diagnosed aggressive periodontitis were recruited from the Department of Periodontology, Faculty of Dentistry, Tabriz University of Medical Sciences. The selected patients had a minimum of 12 teeth with at least 3 teeth in each quadrant with ≥4 mm of probing depth.

The examiner evaluated the pocket depths and clinical attachment levels on two occasions 48 hours apart, and data were analyzed with Student’s t-test. Calibration was validated because evaluations were not significantly different between the two occasions (P>0.05).

Exclusion criteria consisted of periodontal treatment and antibiotic use within the last 6 months, systemic disease affecting periodontal status, smoking and pregnancy.

All the patients were informed about the study and submitted their informed consent for 3 months during the study. The study was approved by the Ethics Committee of Tabriz University of Medical Sciences (IRCT Reference number IRCT201211277128N3).

### Clinical Evaluation

The study was performed using the split-mouth design. Clinical parameters of each patient were measured at baseline and 3 months after periodontal treatment. Probing depth (PD), clinical attachment level (CAL), gingival recession (GR), bleeding on probing (BOP), plaque index (PI) and gingival index (GI) were documented by a blind experienced examiner who was not involved in the treatment procedures of the patients. For probing measurements, a manual periodontal probe (UNC-15, Hu-Friedy Co., Chicago, IL, USA) was used. PD was measured in mm from the gingival margin to the base of the pocket. Gingival recession (REC) was recorded in mm from the cemento-enamel junction to the gingival margin at the experimental sites. CAL was calculated as distance in mm from a fixed reference point (as cemento-enamel junction) to the bottom of the pocket. Full-mouth plaque score, referred to as PI, was recorded as the percentage of tooth surfaces that exhibited the presence of plaque detected by the use of a periodontal probe, modified from O’Leary et al.^27^ GI was record for each tooth as described by Loe et al.^28^

### Microbiological Evaluation

At baseline and 3 months after treatment, subgingival plaque samples were collected from the deepest site of the test and control teeth in each patient. After removal of supragingival plaque and calculus with a sterile periodontal curette, each selected site was dried and isolated from saliva with cotton rolls. Then subgingival plaque samples were collected using sterile paper points (#50), carefully inserted into the depth of the pocket from the apical aspect of pockets and kept in position for 15 seconds. The paper cone of each site was inserted in a sterile transport vial and sent to a laboratory for DNA analysis with real-time PCR with a commercially available kit (Primer Design ^TM^ Genesig Kit, Primer Design Ltd, London, UK). In cases of bleeding during removal of supragingival deposits or subgingival sampling, the microbial sampling was postponed to the next session.

The analysis was performed to identify and quantify *A. actinomycetecommitans.* The microbiological analysis was performed as follows by an operator blinded to the study design.

The microbiological analysis consisted of genomic DNA extraction using the Amplisens DNA extraction kit (AmpliSens^®^, Central Research Institute for Epidemiology, Moscow, Russia). In order to quantify the bacterial species in question in the samples, quantitative real-time PCR was used with the Primer Design (Primer Design^TM^ Genesig Kit, Primer Design Ltd, London, UK) using Taqman method in which 3' and 5' ends of the probe were labeled with FAM and TAMRA according to manufacturer’s instructions. Bacterial quantitative real-time PCR amplification protocols consisted of initial hot start at 95°C for 10 minutes for enzyme activation, followed by 50 PCR cycles at 95°C for 10 seconds for denaturation and 60°C for 60 seconds for annealing and extension, with fluorescence emissions monitored during the extension step. Data were analyzed using ABI (Applied Biosystem) software. Standard curves were analyzed by comparing the universal primer set against a serial dilution of A. actinomycetecommitans genomic DNA. Based on the results obtained from the quantitative real-time PCR, the detection frequency of bacterial species in subgingival plaque was calculated.

### Treatment

All the patients received scaling and root planing (SRP) using a piezoelectric ultrasonic (Varios 970, iPiezo Engine^®^ Kanuma Shi, Tochigi, Japan) handpiece with the same-shape tip by the same clinician. The teeth of one quadrant of each arch with ≥4 mm of PD, selected randomly, were additionally treated with PDT. The control group consisted of the selected teeth of contralateral quadrant. Scaling and root planing was terminated when the operator judged the debridement to be adequately performed.

PDT was performed with a diode laser (HANDY Laser, USA, FDA approved) with a wavelength of 670-690 nm and a power of 75 mW for 2 minutes in the test group after application of toluidine blue photosensitize dye (Sigma Chemical Co., St Louis, MO) with an insulin needle, starting from the bottom of the pocket to coat the root surface. After 1 minute, the teeth were rinsed with distilled water to remove excess photosensitizer. Then the pockets were exposed to the laser light for 2 minutes. All the selected tooth surfaces were treated with PTD. Laser application was carried out circumferentially around each tooth. Only proximal surfaces were considered for the clinical measurement. During laser application, the operator and the patient wore eyeglasses for protection. Laser application was performed by an experienced operator who was not involved in the periodontal treatment of patients.

Upon completion of the study all the subjects received full care and then continued with their individualized maintenance program.

### Statistical Analysis

Statistical analysis was performed with the available software (SPSS Inc. Chicago, IL, USA). Primary clinical outcome variable was changes in CAL. The sample size was calculated based on the CAL, considering a 5% alpha error. Mean values and standard deviations (mean ± SD) for the clinical variables were calculated for each treatment modality, based on the subject as the statistical unit. Independent t-test was employed for continuous variables (clinical measurements) after normal distribution of data was confirmed. Paired sample t-test was used within each group before and after treatment. Statistical significance was defined at P<0.05.

## Results

Twenty-four patients completed the course of the study. No complications, such as pain or infection, were observed in this study. All the clinical measurements were carried out in the interproximal region. The clinical measurements of CAL, PD and REC in the test and control groups at baseline and at 3 months are shown in Table 1. A significant reduction in PD and CAL was observed in the two groups after treatment (P<0.05). REC was similar in the SRP group but did not show any significant difference in the test group after 3 months (P>0.05, Table 1). There were no significant differences between the two groups after 3 months (P>0.05).

**Table 1:** Clinical measurements of clinical attachment level (CAL), probing depth (PD) and gingival recession (REC) in the test and control groups at baseline and after 3 months

Clinical measurements	Groups	Baseline	3 Month	P value (Paired samples t-test)
CAL (mm)	PDT	6.58 ± 0.83	5.29 ± 1.26	0.002
	SRP	6.25 ± 1.07	5.50 ± 1.18	0.002
	P (Independent t-test )	0.23	0.558	
PD (mm)	PDT	5.79 ± 1.06	4.29 ± 0.95	0.000
	SRP	5.45 ± 0.77	4.54 ± 0.88	0.000
	P (Independent t-test )	.22	.351	
REC (mm)	PDT	0.83 ± 0.81	1.04 ± 0.90	0.285
	SRP	0.83 ± 0.81	1.25 ± 0.89	0.022
	P (Independent t-test )	1.00	0.428	
GI	PDT	2.54 ± .65	1.17 ± 0.56	0.000
	SRP	2.42± 0.71	1.42 ± 0.58	0.000
	P (Independent t-test )	0.138	0.532	
BOP	PDT	91.70	75.00	0.009
	SRP	100.00	37.50	0.000
	P (Independent t-test )	0.149	0.006	
PDT: Photodynamic Therapy; SRP: Scaling and root planing.				

BOP, PI and GI showed significant decreases after 3 months (P<0.05) but no statistically significant differences were observed in PI and GI between the two groups after treatment (P>0.05). BOP showed a significant decrease in the control group compared to the test group (P<0.05).

**Figure 1.**
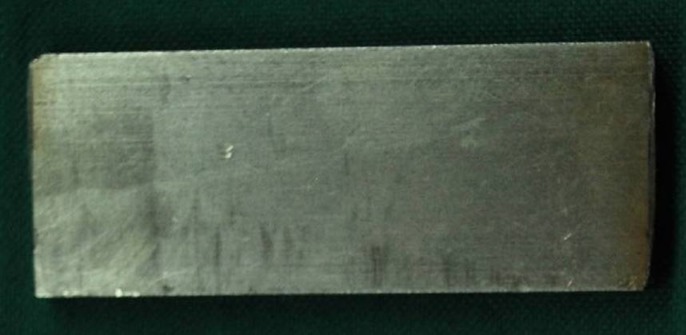
*A. actinomycetecommitans* counts at baseline and after 3 months.

In both groups a significant reduction was seen in *A. actinomycetecommitans* counts after 3 months. However, no significant differences were observed between the two groups (P=0.138) (Figure 1).

## Discussion

As described in a wide range of classic studies, scaling and root planing (SRP) decrease the clinical parameters of periodontitis.^29^ The results of this study also showed this fact in patients with aggressive periodontitis. Nevertheless, additional application of PDT after scaling and root planing did not result in improvements in clinical parameters.

The treatment of aggressive periodontitis remains a challenge for clinicians.^30^ Apart from mechanical debridement of calculi and deposits on root surfaces of teeth, antimicrobial chemotherapy may prevent further periodontal breakdown by pathogens. Numerous systemic and local antimicrobial agents have been evaluated for the treatment of aggressive periodontitis.^30-32^ An obvious advantage of local delivery of antibiotics over systemic administration is that it minimizes disruption of normal microflora of oral and other body sites. However, one problem of this method is difficulty maintaining the therapeutic agent for sufficient time due to elimination of the agents with gingival crevicular fluid.^33^ To overcome this problem, the insertion of fibers or strips that slowly release agents into periodontal pocket has been used.^34^ In addition, the results of studies are uncertain about which antimicrobial agent, dose and duration of supplication yield optimal clinical and antimicrobial effects in these patients. Additionally, bacterial resistance to antibiotics may be the cause of the lack of efficacy of such drugs in the treatment of periodontitis.^35^

The use of PDT, however, is not restricted by such problems as the photosensitizer needs to be retained in the periodontal pocket for only a short time, which may be minutes or seconds, depending on the power of the laser light delivery system used. For example, the results of an animal study on rats showed that 1 mg of toluidine blue per mL was sufficient to reduce the number of viable *P. gingivalis* to below detectable levels by using a light exposure period of only 60 seconds.^36^

The results of this study were consistent with those of some other clinical trials.^37-39^ The results did not exhibit statistically significance differences in clinical parameters like PD, CAL, and REC after treatment of patients with chronic periodontitis with PDT. Another study compared SRP with PDT in patients with aggressive periodontitis^4^ in a split-mouth design; clinical parameters decreased after 3 months, although the results did not reveal significant differences between the two groups. These results are similar to those of the present study. However, a main distinction in that study compared to this study was lack of mechanical debridement before PDT. In the present study, PDT was used as adjunctive treatment after SRP. Without SRP clinicians expect calculus to remain on the root surface, which may serve as a niche for periodontopathogens.^40^

One unexpected finding in this study was that BOP showed a significant decrease in the control group. This finding is difficult to interpret compared to other studies^40,41^ in which reduction in BOP with PDT improved significantly compared to SRP. However, one study^42^ showed improvements in both PDT and SRP groups, with lower values in the PDT group compared to the SRP group.

In this study, the effect of single sessions of PDT on improvement of clinical parameters was evaluated; another study evaluated the effect of five session of PDT. In that study, sites treated with PDT, which had PD of ≤5 mm, showed greater reduction in CAL, PD and BOP.^43^

The results of this study did not show significant differences between the test and control groups in the quantitative evaluation of periodontopathogen *A. actinomycetecommitans* by real-time PCR method. However, a significant decrease was seen in both the test and control groups before and after treatment of periodontitis by PDT after SRP or SRP alone. These results are consistent with those of another study in which PCR was used for evaluation of a wide range of periodontopathogens, e.g. red complex, green complex and *A. actinomycetecommitans.*^37^ In that study *A. actinomycetecommitans* did not show a significant decrease between two groups of SRP and PDT after 3 and 6 months. However, some other bacteria showed significant differences between the test and control groups.

The results of a published in vitro study showed that the ability of laser light to kill periodontal pathogens is species-dependent.^44^ *A. actinomycetecommitans* is more resistant than *P. gingivalis.* A recent study showed a significant decrease in *A. actinomycetecommitans, P. gingivalis,* and *T. forsythensis* counts after 90 days in patients treated with PDT in combination with SRP compared to SRP alone.^39^ The results of other studies have indicated that PDT and SRP affect different bacterial species, with PDT being effective in reducing *A. actinomycetemcomitans* counts compared to SRP. On the other hand, SRP was more efficient than PDT in reducing periodontal pathogen counts of the red complex.^45^ These results are not consistent with those of this study in term of the number of *A. actinomycetemcomitans* bacterial species. It is difficult to interpret this controversy.

In another study, it was suggested that PDT may still bring some possible benefits, such as an additional effect at sites with difficult access (e.g. furcations, concavities), influencing the biofilm in residual deep pockets.^46^ In this study all the measurements were carried out in interproximal areas.

In this study, toluidine blue was used as a photosensitizer, which has been extensively tested in vitro and in vivo.^10,36,47,48^ Toluidine blue has been shown to be highly effective when used with a soft laser irradiation.^10,11,49^

In this study, the microbial tests were taken only at baseline and 3 months after SRP. It is obvious that the effect of one-session PDT cannot sustain for 3 month. This may be one of the limitations of this study.

This study was designed as a split-mouth study. The attractiveness of the design is that it eliminates a lot of inter-individual variability from the estimates of treatment effects. In addition, in this type of study, the treatment of one site of the mouth may affect other sites,especially when microbiological evaluations should be performed.

In split-mouth-design studies, many trials have failed to take advantage of research designs in statistical analysis of data. In addition, very few studies have considered the possibility of order effects or reduced bias through blinding procedures. In this study the operator of treatment and the examiner were blinded to the study design.^50^

## Conclusion

Within the limitations of this study, including short follow-up period, the results did not show additional benefits from PDT as an adjunctive treatment of patients with aggressive periodontitis. Other studies may be required for patients with aggressive periodontitis during the maintenance of treatment outcomes. Evaluation of areas with difficult access, like furcation areas, may be beneficial in other study designs.
